# Rubber tail illusion is not obvious in SH3 and multiple ankyrin repeat domains 3 (*Shank3*)-knockout mice

**DOI:** 10.1093/oons/kvag014

**Published:** 2026-09-21

**Authors:** Makoto Wada, Yo Shinoda, Shigeo Uchino

**Affiliations:** Department of Rehabilitation for Brain Functions, Research Institute of National Rehabilitation Center for Persons with Disabilities, Namiki 4-1, Tokorozawa, Saitama 359-8555, Japan; Department of Rehabilitation for Sensory Functions, Research Institute of National Rehabilitation Center for Persons with Disabilities, Namiki 4-1, Tokorozawa, Saitama 359-8555, Japan; Laboratory of Environmental Health, School of Pharmacy, Tokyo University of Pharmacy and Life Sciences, Horinouchi 1432-1, Hachioji, Tokyo 192-0392, Japan; Department of Integrated Science and Engineering, Faculty of Science and Engineering, Teikyo University, Toyosatodai 1-1, Utsunomiya, Tochigi 320-8551, Japan

**Keywords:** body image, autism spectrum disorder, mouse model, body ownership illusion, Shank3, rubber tail illusion

## Abstract

Atypical body representations are often observed in individuals with autism spectrum disorder (ASD). The rubber tail task, which involved stroking both a real tail and a rubber tail, was designed to investigate the body ownership illusion in mice. A previous study showed that the body ownership illusion (i.e. the rubber tail illusion) is not obvious in Ca^2+^-dependent activator protein for secretion 2 (*Caps2*)-knockout (KO) mice, which exhibit ASD-like phenotypes. If this phenotype is a widespread feature of ASD models, then the rubber tail illusion should be attenuated in other strains of ASD model mice. Therefore, we investigated the rubber tail illusion in SH3 and multiple ankyrin repeat domain 3 (*Shank3*) KO mice, a common ASD mouse model. After synchronous stroking of the real and rubber tails, a significantly stronger response was observed in wild-type (WT) mice when the rubber tails were grasped. In contrast, no significant difference in the response rate was observed between synchronous and asynchronous stroking in *Shank3*-KO mice, although the response rate was much lower than that in WT mice. These results suggested that the rubber tail illusion was not obvious in *Shank3*-KO mice, although the influence of hyporeactivity cannot be completely ruled out.

## Introduction

Atypical body representations are often observed in individuals with autism spectrum disorders (ASD). For instance, the body ownership illusion does not typically occur in individuals with ASD (Cascio et al. 2012, Paton et al. 2012). In the rubber hand illusion (RHI), one of the most well-known body ownership illusions, neurotypical participants perceive the rubber hand as becoming their own hand when their hand and the rubber hand are synchronously stroked with brushes. (Botvinick and Cohen 1998, Ehrsson 2012, Tsakiris et al. 2010, Tsakiris and Haggard 2005). In this task, the integration of simultaneous visual and tactile stimuli changed the subjective location of the tactile stimuli to a rubber hand, creating the sensation of owning the hand. In contrast, Cascio et al. (2012) reported that children with ASD initially found it difficult to experience the RHI, but after more than 5 minutes, they began to experience the illusion. Therefore, RHI occurs later in individuals with ASD than in those without. Similarly, Paton et al. (2012) suggested that people with ASD exhibit more precise proprioception during the RHI, resulting in decreased sensitivity to visuotactile-proprioceptive discrepancies. Greater reliance on proprioception, also reported in other tactile or motor tasks (Haswell et al. 2009, Wada et al. 2014), is thought to inhibit body ownership illusions in people with ASD. Consequently, individuals with ASD exhibit stronger associations between touch and proprioception than between vision and touch. Animal models, such as ASD-related gene knockout (KO) mice, should be used to elucidate the neural basis of these atypical body representations in people with ASD.

Previous studies have reported the occurrence of the ‘body ownership illusion’ in both non-human primates (Shokur et al. 2013) and rodents (Buckmaster et al. 2020, Wada et al. 2019, Wada et al. 2016). The rubber tail task for mice was designed to evaluate atypical body representations. In this task, synchronous strokes were applied to a real tail and a rubber tail using two small brushes (Wada et al. 2016). The mice responded as if their tails were being touched when their rubber tails were grasped (the rubber tail illusion in mice). The rubber tail illusion may be caused by visuotactile integration. In a previous study, the rubber tail illusion was not obvious in Ca^2+^-dependent activator protein for secretion 2 (*Caps2*)-KO mice (Wada et al. 2019), which exhibit autistic-like phenotypes. Another study suggested that c-Fos expression in the higher sensory cortex, where multisensory signals are integrated, decreased in *Caps2*-KO mice during a task (Wada et al. 2021). The weakened rubber tail illusion may have been caused by deficits in visuotactile integration during this task. Additionally, a recent study reported that *Caps2*-KO mice have decreased plasma oxytocin levels despite increased hypothalamic and pituitary oxytocin levels, suggesting insufficient oxytocin release (Fujima et al. 2021). Therefore, the weakened rubber tail illusion in *Caps2*-KO mice may be related to oxytocin dysfunction. This is because the body ownership illusion is associated with empathy (Asai et al. 2011) and oxytocin levels (Crucianelli et al. 2019, Ide and Wada 2017, Spengler et al. 2019). However, it is unclear whether the weakened rubber tail illusion observed in *Caps2*-KO mice is specific to this strain due to insufficient oxytocin release or whether it is widespread in ASD mouse models. Investigating whether the rubber tail illusion is weakened in other ASD mouse strains could help reveal the neural basis of this phenomenon.

Among the various ASD-related genes, SH3 and multiple ankyrin repeat domains 3 (*Shank3*) are among the best-known (Uchino and Waga 2013). It is mainly expressed at the synapses of excitatory neurons. Shank3 acts as a scaffolding protein that interacts with various synaptic molecules (Naisbitt et al. 1999, Uchino and Waga 2013). This protein plays important roles in the formation, maturation, and maintenance of synapses. Mutations in the *Shank3* gene have been identified in some individuals with ASD, *Shank3*-KO mice also show ASD-like behaviours, including impairments in social interaction and repetitive behaviours (Bozdagi et al. 2010, Uchino and Waga 2013, Wang et al. 2011). For example, male *Shank3*-KO mice exhibit fewer social sniffs and ultrasonic vocalisations when interacting with females in oestrus (Bozdagi et al. 2010). Another study suggested that *Shank3*-KO mice exhibited atypical social behaviours, atypical communication patterns, increased repetitive behaviours, and impaired learning and memory (Wang et al. 2011). Therefore, *Shank3* is thought to be strongly involved in the development of ASD.

Regarding *Shank3*-KO mice, a variety of sensory atypicalities have been reported in addition to core features, such as impairments in social communication and repetitive behaviour (Chen et al. 2020, Han et al. 2016). For instance, pain hyperalgesia, which may be mediated by the transient receptor potential vanilloid 1 (TRPV1) pathway in the peripheral nervous system (Tominaga et al. 2001), is reduced in *Shank3*-KO mice (Han et al. 2016). In contrast, hyperreactivity to tactile stimuli has been reported in *Shank3*-KO mice, which may be caused by dysfunction of cortical GABAergic neurons (Chen et al. 2020). ‘Hypesthesia to pain’ and ‘hypersensitivity to tactile stimuli’ are sensory features commonly observed in individuals with ASD, and *Shank3*-KO mice appear to exhibit these sensory characteristics.

Therefore, it is intriguing to examine the rubber tail illusion in *Shank3*-KO mice, which have atypical neural circuits in the brain, including the sensory system. This contrasts with *Caps2*-KO mice, which exhibit dysfunction in oxytocin release, a process important for empathic systems and embodiment. In the present study, we investigated whether the rubber tail illusion is attenuated in *Shank3*-KO mice (Experiment 1) and confirmed their basic responses to tail tactile stimulation (Experiment 2).

## Methods

### Animals

*Shank3*-KO (n = 12) and WT (n = 13) male mice were used in this study. All WT mice were the littermates of *Shank3*-KO mice (Uchino and Waga 2015). Mice were transported from the Tokyo University of Pharmacy and Life Sciences, where breeding and genotyping were conducted, to the Research Institute of National Rehabilitation Center for Persons with Disabilities, where behavioural testing was conducted, using specialised animal carriers (Sankyo Labo Service Corporation, Inc., Tokyo, Japan). Littermates were group-housed (2–4 per cage) under controlled conditions (approximately 23°C, 30–50% humidity, 12 h light/dark cycle). Behavioural tests were performed during the light phase in drug- and surgery-naive mice. Prior to the experiments, the mice were handled by the experimenter for at least 3 days. Behavioural testing commenced when the mice were 9–11 weeks old. Animal handling and experimental scheduling were performed in accordance with previously described protocols (Wada et al. 2019). The present research, including experimental protocols, was reviewed and approved by the institutional ethics committee for animal experimentation. All experiments were conducted in accordance with the relevant regulations and guidelines of the Ministry of Health, Labour, and Welfare of Japan and complied with the recommendations in the ARRIVE guidelines.

In this study, male mice were used for behavioural assessments to avoid the influence of the estrous cycle as an initial step. As trends among females were not investigated, this would be a limitation to generalisability; therefore, future research needs to include assessments involving both sexes.

### Apparatus

**Figure 1:**
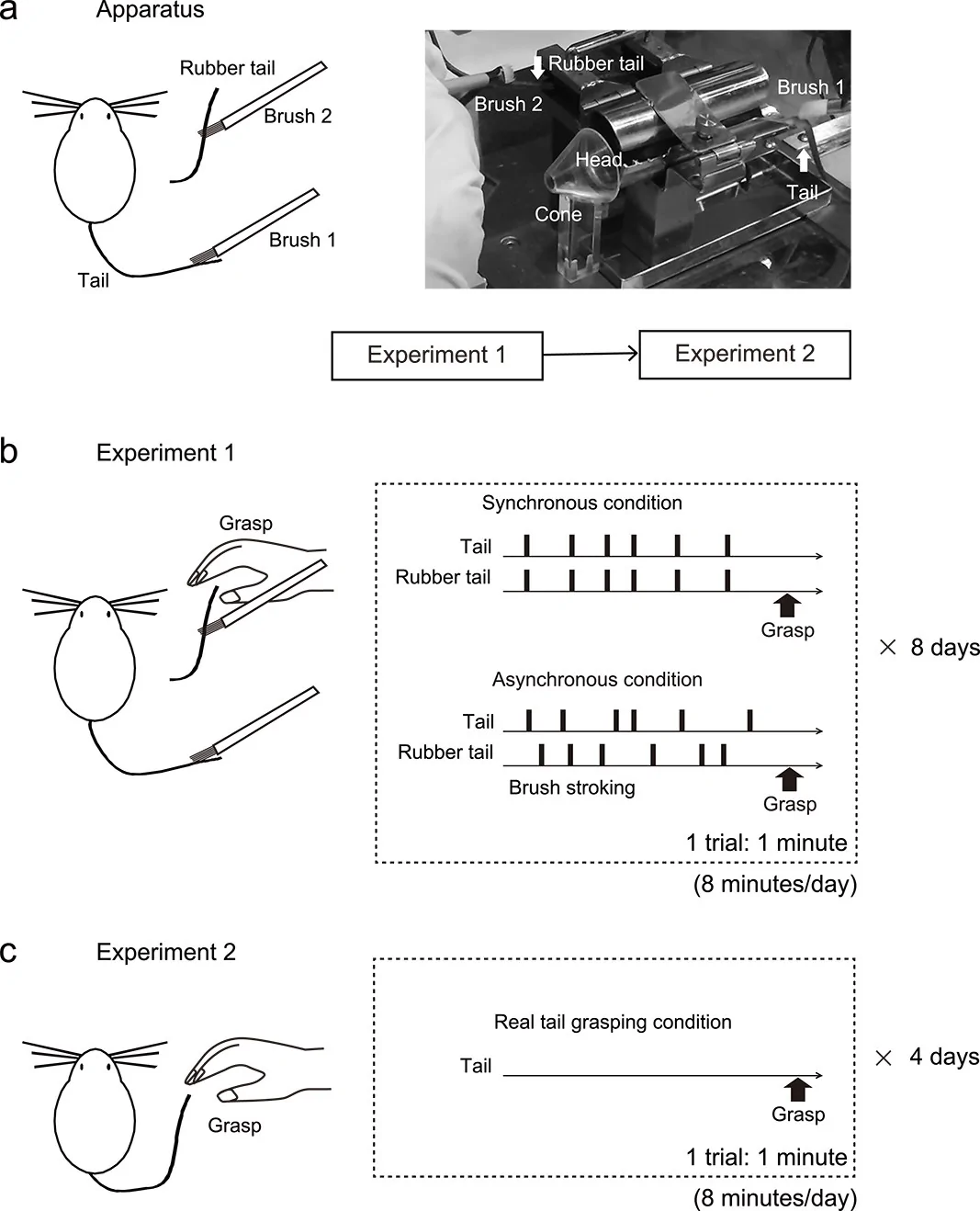
Rubber tail task for mice. (a) Apparatus: A tail and rubber tail received brush stroking in a stainless-steel tube, as shown in the right panel. one end of the tube was open, and the other was connected to a transparent plastic cone. A rubber tail was placed either on the right or left of the cone. (b) Experiment 1: Rubber tail task (synchronous vs asynchronous conditions). In the synchronous condition, both *Shank3*-KO and WT mice received synchronous stroking to both real and rubber tails using two small brushes for 1 min (0.5–2 Hz). As a control, they received asynchronous stroking for 1 min (0.5–2 Hz) in the asynchronous condition. Details of the apparatus are identical to those in our previous study (Wada et al. 2019, Wada et al. 2016). (c) Experiment 2: Real tail grasping task. In the task, responses to real tail grasping without any stroking were examined. Experiment 2 was conducted after Experiment 1.

Mice were tested in an open-ended stainless-steel tube (O’Hara & Co., Ltd., Tokyo, Japan). The apparatus (Fig. 1a) was designed for the rubber tail task as previously described (Wada et al. 2019, Wada et al. 2016). Prior to behavioural testing, the mice were trained to remain in the tube with their heads stationary for >10 min in each cage. During training, if a mouse jumps out of the tube, it was promptly returned. The number of days for initial training was 7.88 ± 2.05 days.

### Experiment 1: Rubber tail task

To evaluate body ownership illusion in the ASD mouse model, we investigated whether the rubber tail illusion was weakened in *Shank3*-KO mice. After initial training to remain in the tube in the rubber tail task (Fig. 1b), mice (*Shank3*-KO: n = 12; WT mice: n = 13) underwent 8-min daily tests under two conditions: 1) synchronous stroking of the real tail and a rubber tail (synchronous condition), and 2) asynchronous stroking of both tails (asynchronous condition), each with two small brushes. Thus, a total of 16 minutes of daily tests were conducted, and the order of conditions was counterbalanced between sessions. The experimenter, who was blinded to the experimental group, manually stroked the actual and rubber tails using brushes (0.5–2 Hz) for approximately 0.5–1 sec. After approximately 1 min of stroking, the researcher grasped the rubber tail. The rubber tail task was conducted for eight days.

### Experiment 2: Real tail grasping task

In the real tail grasping task (Fig. 1c), we investigated the reactivity to the real tail without brush stroking. In this task, the response to real tail grasping was tested in a subset of mice (10 *Shank3*-KO and 11 WT) after Experiment 1 (rubber tail task). Two mice from each group were not included in Experiment 2 because they were reserved for a separate preliminary study, and their selection was unrelated to the behavioural results of Experiment 1. The tail of each mouse was placed to the right, without a rubber tail, and held with surgical tape. During the 8 min test, the tail was grasped at approximately 1 min intervals. The test was conducted for four days.

### Analysis and statistics

Head movements of the mice were recorded using a digital video camera (GZ-RX680-B, JVC KENWOOD Corporation, Yokohama, Japan) and evaluated by a technical staff member blinded to the experimental conditions and groups. According to a previous study (Wada et al. 2016), if the head immediately turned toward the rubber tail or retracted into the tube, it was rated as a full response (1.0). If the response was relatively short or delayed (approximately 1 s), it was considered an intermediate response (0.5). When the head moved only slightly, the score was set to 0.25. In addition, trials in which the mice did not respond were assigned a score of zero (0). The scores were averaged for each condition.

Among the response rate data, a normality test (Shapiro–Wilk test) was conducted. Statistical comparisons were performed using a paired *t-*test (between conditions) or a Welch’s *t-*test (between groups). Statistical analyses were performed using MATLAB (MathWorks, Natick, MA, USA), R (4.1.0 GUI 1.76), and G^*^Power (version 3.1.9.6).

### Histology

Regarding immunostaining, the sample size was reduced owing to the retention of several mice in other studies and the loss of specimens resulting from technical failures during perfusion or sectioning. Finally, 12 WT mice and 8 *Shank3*-KO were used for further histological analysis. After the behavioural test, the mice were immediately anaesthetised using isoflurane, given an overdose of pentobarbital intraperitoneally, and perfused intracardiacally with approximately 60 ml of saline, followed by approximately 30 ml of 4% paraformaldehyde in 0.1 M phosphate buffer (obtained from Wako Junyaku, Osaka, Japan). The brains were then extracted and stored in 4% paraformaldehyde at 4°C for 12 hours, and then transferred to 30% sucrose in 0.1 M phosphate buffer at 4°C until they sank. The brain blocks were mounted in OCT compound (Miles Inc., Elkhart, IN, United States), snap-frozen in a pentane-hexane (1:1) bath (UT-2000F, Tokyo Rikakikai Co., Ltd., Tokyo, Japan) at −100°C, and stored at −80°C. Coronal brain slices (16 μm) were cut on a freezing microtome (CM3050S, Leica, Nussloch, Germany) and were mounted on pre-coated slides (MAS-coated slide, Matsunami Glass Ind., Ltd., Osaka, Japan). Histological experiments were performed in accordance with previously described protocols (Wada et al. 2021).

To detect neuronal cells, immunostaining was performed with an anti-neuronal nuclei (NeuN) antibody (A60 biotin-conjugated; Sigma-Aldrich). The sections were incubated for 5 min at 90°C in 0.01 M citric acid (pH 6.0) to retrieve antigens. The sections were then incubated for 1 h in 3.6% MOM blocking reagent (Mouse-on-Mouse Immunodetection Kit, Vector Laboratories). The sections were then incubated for 5 min in MOM diluent (Mouse on Mouse Immunodetection Kit; Vector Laboratories) and then incubated for 30 min at room temperature in MOM diluent containing the anti-NeuN antibody (1:1000). Sections were incubated for 10 min with a 1∶250 dilution of biotinylated anti-mouse antibody (Mouse-on-Mouse Immunodetection Kit; Vector Laboratories). Immunostaining was conducted using an automated staining device (Histostaner; Nichirei Biosciences Inc., Tokyo, Japan) by contracted companies (Morphotechnology Inc., Sapporo, Japan).

### Comparisons of NeuN positive cell densities

We calculated the densities of NeuN-positive cells and compared them among the groups, as previously described (Wada et al. 2006, 2010, Wada et al. 2021). Images of each section were acquired using a slide scanner with a bright-field microscope (×20, Nano Zoomer S210, Hamamatsu Photonics, Hamamatsu, Japan), calibrated so that 1 pixel corresponded to 2 μm. The section images were scanned using a contract (Morphotechnology, Inc., Sapporo, Japan). For each mouse, neighbouring sections around the somatosensory and motor regions were captured at three levels from bregma (+0.5, −1, and − 2 ± 0.2 mm), according to a mouse brain atlas (Paxinos and Franklin, 2001).

The adjusted image of a whole section was fast Fourier transform (FFT)- band-pass filtered using the NIH ImageJ program (NIH, Bethesda, MD, United States) to eliminate low-frequency drifts (>20 pixels = 40 μm) and high-frequency noise (<1 pixel = 2 μm). The filtered images were processed using a homemade program developed in MATLAB 2014 with the Image Processing and Statistics toolboxes (MathWorks Inc., Natick, MA, United States). NeuN-positive cells were detected by recording the centroid positions of areas of constant size that stained darker than the threshold (1–80 pixels). The NeuN positive cell density map was prepared for each section by calculating the number of the immunostained cells in each compartment sized 120 μm × 120 μm (60 pixel × 60 pixel), and the map was resized and normalised to a standard section (150 × 200 pixels, 1 pixel ≒ 50 μm). Matrices were created by piling maps for each group.

Using these matrices, a pixel-by-pixel between-group comparison of neuronal cell densities in each matrix was performed. *T*-test was repeatedly applied to each block, after spatially smoothing each map with a Gaussian filter (SD: 8 pixels ≒ 400 μm). We identified the regions in which the WT mice (*n* = 12) showed higher (or lower) NeuN cell density than the *Shank3*-KO mice exposed to synchronous conditions (*n* = 8). The *p-values* from the t-test at each point (p < 0.05) were mapped to show areas indicating differences between groups.

## Results

In the present study, we investigated whether the rubber tail illusion (Wada et al. 2016) occurs in *Shank3*-KO and WT mice.

### Rubber tail illusion was weakened in *Shank3*-KO mice

During the initial training, the mice were trained to remain in the tube for several minutes. In the rubber tail task (Experiment 1), mice underwent daily tests for 8 min under two conditions using two small brushes (Fig. 1b). The mouse received synchronous stroking of the actual and rubber tails in the synchronous condition and asynchronous stroking of both tails in the asynchronous condition. After the tail was stroked for approximately 1 min, an experimenter who was blinded to the experimental groups grasped the rubber tail, and the response was recorded.

**Figure 2:**
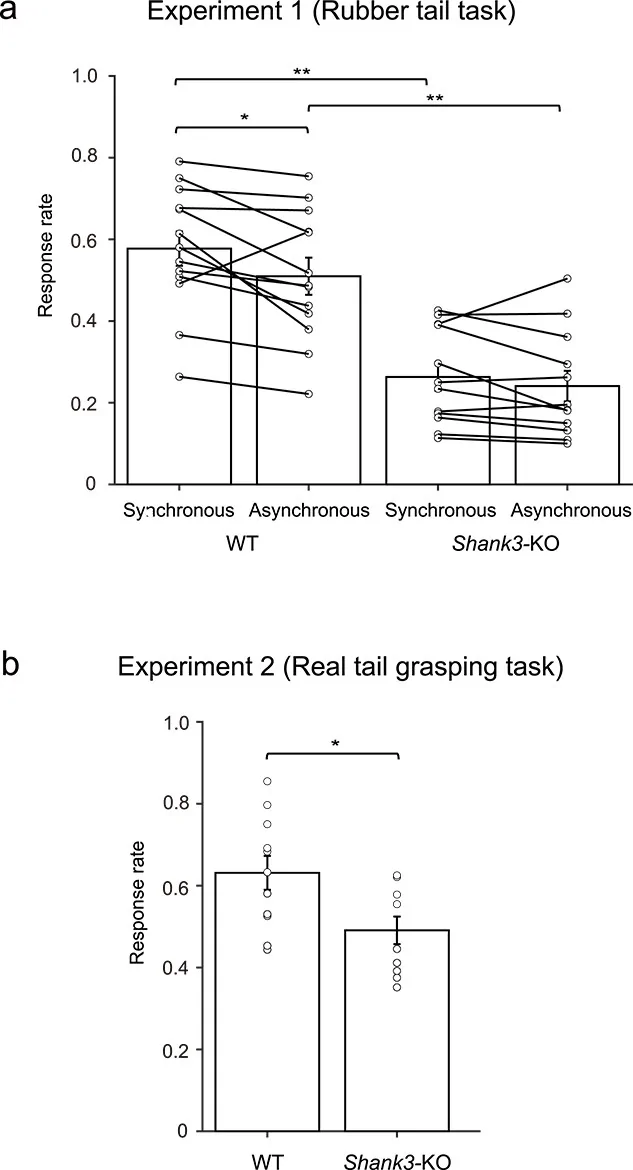
Response rates for the behavioural task in *Shank3*-KO and WT mice. (a) Response rates in the rubber tail task (experiment 1). Bar graphs indicate averaged values from 13 WT mice and 12 *Shank3*-KO mice. Each marker indicates the average response for each mouse in each condition. The response rates for the WT mice were significantly higher in the synchronous condition, while there was no significant difference between the response rates of synchronous and asynchronous conditions in *Shank3*-KO mice (^*^p < 0.05, ^**^p < 0.01). The results indicate that the rubber tail illusion was not obvious in the *Shank3*-KO mice. (b) Real tail grasping task (experiment 2). Each bar indicates the mean response rate of four sessions per mouse for each group (WT mice: n = 11, *Shank3*-KO mice: n = 10). The mean response rate was significantly lower in *Shank3*-KO mice than in WT mice (^*^p < 0.05).

The responses from the eight sessions (days) were averaged for each condition. Individual data are presented in Supplementary Tables 1 and 2. The averaged response rates for WT mice in both conditions were 0.58 ± 0.042 (synchronous condition) and 0.51 ± 0.044 (asynchronous condition; mean ± standard error), respectively. The averaged response rates for *Shank3-*KO mice in both conditions were 0.26 ± 0.034 and 0.24 ± 0.037, respectively (Fig. 2a).

After testing for normality in both the synchronous (WT: *W* = 0.96, *p* = 0.75; *Shank3*-KO: *W* = 0.90, *p* = 0.14) and asynchronous conditions (WT: *W* = 0.98, *p* = 0.96; *Shank3*-KO: *W* = 0.90, *p* = 0.17) using the Shapiro–Wilk test, the following comparisons were conducted. For the WT group, the response rate was significantly higher in the synchronous condition than in the asynchronous condition (*t_12_* = 2.73, *p* = 0.0183 < 0.05/2, 95% *confidence interval* (CI) = [0.0136, 0.122], Cohen’s *d* = 0.76, paired *t*-test with the Holm correction, Fig. 2a), which is in line with previous studies (Wada et al. 2019, Wada et al. 2016). In contrast, the difference in the response rate was not significant between the synchronous and asynchronous conditions (*t_11_* = 1.30, *p* = 0.222 > 0.05, 95% CI = [−0.0152, 0.0587], Cohen’s *d* = 0.37, paired *t*-test with the Holm correction) for *the Shank3-*KO group. The group differences were compared for each condition. Under both conditions, the response rate was significantly higher in the WT group than in the *Shank3-*KO group (*t_22.3_* = 5.84, *p* < 0.00001 < 0.05/4, 95% CI = [0.20, 0.43], Cohen’s *d* = 1.51; *t_22.7_* = 4.68, *p* = 0.000107 < 0.05/3, 95% CI = [0.150, 0.388], Cohen’s *d* = 1.36, Welch’s *t*-test with the Holm correction, respectively). In *Shank3*-KO mice, the rubber tail illusion was not obvious, although the response rates were generally low in *Shank3*-KO mice.

When the data from the eight days were analysed by dividing them into the first and second four-day periods (Supplementary Tables 3 and 4), no significant differences in response rates were observed between the two periods for either WT or *Shank3*-KO mice under either the synchronous (WT: *t_12_* = 0.37, *p* = 0.72, 95% *confidence interval* (CI) = [−0.060, 0.084], Cohen’s *d* = 0.10; *Shank3*-KO mice: *t_11_* = −0.94, *p* = 0.37, 95% *confidence interval* (CI) = [−0.10, 0.041], Cohen’s *d* = −0.27, paired *t*-test) or asynchronous conditions (WT: *t_12_* = −0.41, *p* = 0.69, 95% *confidence interval* (CI) = [−0.082, 0.056], Cohen’s *d* = −0.11; *Shank3*-KO mice: *t_11_* = −0.60, *p* = 0.56, 95% *confidence interval* (CI) = [−0.087, 0.050], Cohen’s *d* = −0.17, paired *t*-test). Therefore, in the present conditions, the effects of habituation or learning were not significant.

### Responses for real tail grasping were weakened in *Shank3*-KO mice

The responses to real tail grasping were investigated in a subset of mice (Fig. 1c). Under the task (Experiment 2), the tail of each mouse was placed on the right side and held with surgical tape. During the 8-min test, the actual tail was grasped by an experimenter at approximately 1-min intervals, as in a previous study (Wada et al. 2019). When the actual tail was grasped in both WT mice (n = 11) and *Shank3*-KO mice (n = 10), similar but slightly higher responses were observed in WT mice than in *Shank3*-KO mice (Fig. 2b, WT: 0.63 ± 0.041; *Shank3*-KO: 0.49 ± 0.033). The Shapiro–Wilk test did not indicate rejection of normality for the response rates in the respective groups (WT: *W* = 0.96, *p* = 0.75; *Shank3*-KO: *W* = 0.88, *p* = 0.14). The response rate was significantly higher in the WT group than that in the *Shank3-*KO group (*t_18.6_* = 2.63, *p =* 0.0167 < 0.05, 95% CI = [0.0285, 0.252], Cohen’s *d* = 1.00, Welch’s *t*-test). Individual data are presented in Supplementary Tables 1 and 2.

When the data from the four days were analysed by dividing them into the first and second two-day periods (Supplementary Tables 3 and 4), no significant differences in response rates were observed between the two periods for WT mice (*t_10_* = −1.55, *p* = 0.15, 95% *confidence interval* (CI) = [−0.18, 0.032], Cohen’s *d* = −0.47, paired *t*-test), and slightly higher response rate in the second periods was observed in *Shank3*-KO mice (*t_9_* = −2.63, *p* = 0.027, 95% *confidence interval* (CI) = [−0.30, −0.023], Cohen’s *d* = −0.83). The *Shank3*-KO mice, the response tended to be greater during the latter half of the experiment, whereas it was not significant in WT mice. Therefore, habituation did not appear to have occurred under the present condition.

### Neural cell densities in *Shank3*-KO mice

We compared the neuronal density in the sensory-motor cortex between the two groups. Sections of the mouse brain were immunostained with an anti-neuronal nuclei (NeuN) antibody, which can stain the nuclei of neuronal cells, and the NeuN-positive cell densities were automatically counted (Supplementary Fig. 1a). Using the statistical parametric mapping method for immunopositive cell densities (Wada et al. 2006), we compared the neuronal cell density of *Shank3*-KO mice with that of WT mice at the section level (approximately bregma +0.5, −1, and − 2 mm), as shown in Supplementary Fig. 1b. After cluster-level control (> 144.5 pixels), as previously reported (Wada et al. 2021), neuronal cell densities in the bilateral motor cortex (primary/secondary motor cortex) were significantly higher in *Shank3*-KO mice (p < 0.05, bregma +0.5 mm), while those in the hypothalamus were high in WT mice (p < 0.05, bregma +0.5 mm).

## Discussion

In the present study, the response rates in the synchronous condition were higher than those in the asynchronous condition in WT mice, which is consistent with previous studies (Wada et al. 2019, Wada et al. 2016). However, there was no significant difference in response rates between the synchronous and asynchronous conditions in *Shank3*-KO mice in the rubber tail task. These results suggest that the rubber tail illusion was not apparent in *Shank3*-KO mice, as in *Caps2*-KO mice (Wada et al. 2019). In other words, these findings may reflect atypical visual-tactile sensory integration, which might be a feature shared across multiple ASD mouse models rather than specific to a single strain.

In the present study, the finding that the rubber tail illusion was not obvious is consistent with that of a previous study using *Caps2*-KO mice (Wada et al. 2019), whereas *Shank3*-KO mice exhibited a lower overall response. For instance, the response rates of *Caps2*-KO mice were lower than those of WT mice under synchronous conditions. However, no significant differences were observed between the two groups in the asynchronous condition. In contrast, the response rates were lower in *Shank3*-KO mice than in WT mice under both conditions. Furthermore, *Caps2*-KO and WT mice responded similarly to real tail grasping (Wada et al. 2019) whereas *Shank3*-KO mice responded less frequently, although some mice showed responses comparable to those of WT mice (Fig. 2b). The difference in response rates between the two strains (*Caps2*-KO and *Shank3*-KO) in the rubber tail test (Experiment 1) may be attributable to differences in their motor activity. While no differences compared to WT mice have been reported for *Caps2*-KO mice in the open-field test (Sadakata et al. 2007), a significant reduction in the total distance travelled has been reported in *Shank3*-KO mice (Mei et al. 2016). Although both strains share a tendency toward high anxiety (Kabitzke et al. 2018, Sadakata et al. 2007, Shinoda et al. 2011, Song et al. 2019), the hyporeactivity observed in *Shank3*-KO mice was particularly pronounced. Given that reduced responses were observed not only in the rubber tail test (Experiment 1) but also in the reaction to the real tail-grasping task (Experiment 2) in this study, these findings can be interpreted as reflecting a general decline in responsiveness. An overall weak response could affect the results by reducing the magnitude of differences between conditions. However, among the 12 *Shank3*-KO mice, four individuals showed responses in the asynchronous condition that were at least as large as those in the synchronous condition (Supplementary Tables 1 and 2). This is difficult to explain solely by a proportional reduction in overall responsiveness and is consistent with an attenuated rubber tail illusion in *Shank3*-KO mice, because a mere overall reduction in response magnitude is unlikely to alter the relative magnitude relationship.

However, because tactile atypicality (Chen et al. 2020, Han et al. 2016) and atypical stress responses (Lin et al. 2021) have also been reported in *Shank3*-KO mice, the possibility that various factors are involved cannot be ruled out. In humans, a case study reported that RHI is less likely to occur alongside excessive cortical activation in response to tactile stimuli in individuals with neuro developmental disorders (e.g. ADHD) who experience sensory hypersensitivity (Boehme et al. 2020). This is consistent with the interpretation of the enhanced perceptual functioning (EPF) hypothesis in ASD (Mottron et al. 2006). The influence of sensory hypersensitivity on body ownership illusion should be examined in the future.

In this study, the absence of a significant difference in response rates between the synchronous and asynchronous conditions in *Shank3*-KO mice suggests that the rubber tail illusion was not obvious in this strain, as in *Caps2*-KO mice (Wada et al. 2019). Therefore, the atypicality of body ownership illusions in ASD (Cascio et al. 2012, Paton et al. 2012) is partially mimicked in multiple strains of ASD mouse models. In humans, body ownership illusions, including the RHI, are caused by the multisensory integration of simultaneous visuotactile stimuli (Armel and Ramachandran 2003, Botvinick and Cohen 1998, Tsakiris and Haggard 2005). We speculated that visuotactile integration would be atypical in *Shank3*-KO mice, as has been suggested for *Caps2*-KO mice (Wada et al. 2021). Shank3 is a synaptic scaffolding protein whose pathological alterations disrupt the excitatory/inhibitory balance of the brain (Chen et al. 2020, Han et al. 2013). As the excitatory/inhibitory balance of the nervous system is also known to be important for the integration of multisensory signals (Gogolla et al. 2014), this may have contributed to the weakened rubber tail illusion in the current study. Moreover, the involvement of the primary somatosensory cortex during the rubber tail task needs to be considered because significant task-dependent c-Fos expression in the primary somatosensory cortex during the rubber tail task has been reported in WT mice, although not statistically significant differences in c-Fos expression have been reported for these regions in *Caps2*-KO mice (Wada et al. 2021). The excitatory/inhibitory imbalance observed in *Shank3*-KO mice may be widespread in the whole brain because Shank3 acts as a scaffolding protein and interacts with many synaptic molecules (Uchino and Waga 2013). Moreover, previous studies have suggested that the primary somatosensory cortex also receives visual inputs (Masse et al. 2017); therefore, the involvement of the primary somatosensory cortex should be analysed in more detail in future studies.

In addition, we supplementally found that neuronal cell densities in the motor cortex were high in *Shank3*-KO mice, whereas those in the hypothalamus were high in WT mice. Therefore, we found that the density of neurons was higher or lower in different regions than that in WT mice. This diversity in the nervous system may be related to the atypicality of the rubber tail illusion. The findings from NeuN immunolabelling provide a preliminary comparison of neuronal anatomical density but do not directly demonstrate a causal relationship with task performance. As the current results do not demonstrate a link between behavioural outcomes and histological characteristics, further neuroscientific studies are needed to clarify the relationship between these histological findings and behavioural performance.

In the rubber tail task, there was no significant difference in response rates between the synchronous and asynchronous conditions in *Shank3*-KO mice. Although the influence of the overall decline in the response cannot be completely ruled out, the rubber tail illusion was not obvious in *Shank3*-KO mice. We interpreted possible impairments in multisensory integration in the nervous system as related to weakened body ownership illusions. Further studies are required to clarify the neural mechanisms underlying atypical body representation in *Shank3*-KO mice.

## Supplementary Material

SupplementaryData260817_kvag014

## Data Availability

The datasets supporting the conclusions of the present study are included in Supplementary data.
