# Supplementary material for: Rubber tail illusion is not obvious in SH3 and multiple ankyrin repeat domains 3 (Shank3)-knockout mice
Source: Oxf Open Neurosci. 2026 Sep 21;5:kvag014. doi: 10.1093/oons/kvag014 (PMC13613341; doi:10.1093/oons/kvag014)
Supplement: Supplementary file 1 [file supplementarydata260817_kvag014.docx]

**Supplementary Figure 1. Neural cell densities in *Shank3*-KO mice.**

**Supplementary Figure 1. Neural cell densities in *Shank3*-KO mice**. (a) NeuN-positive cell density maps (PCDM). PCDMs of three different levels (bregma +0.5, −1, and −2 mm) are shown in rows for each group (columns: *Shank3*-KO, WT). Each PCDM map was aligned according to the side of the rubber tail and combined. Each boundary was determined by reference to the brain atlas (Paxinos & Franklin, 2001). M2, secondary motor cortex; M1, primary motor cortex; Pir, Piriform cortex, SI, primary somatosensory cortex; S2, secondary somatosensory cortex; MPtA, medial parietal association cortex; LPtA, lateral parietal association cortex; Au1, primary auditory cortex; AuD, secondary auditory cortex dorsal area; AuV, secondary auditory cortex ventral area; V1, primary visual cortex; V2M, medial secondary visual cortex; V2L, lateral secondary visual cortex. Scale bar: 1 mm. (b) Comparison of neural cell densities between *Shank3*-KO and WT mice (columns: *Shank3*-KO > WT, WT > *Shank3*-KO). *Shank3*-KO > WT: Areas that showed significantly higher densities of neural cells in the *Shank3*-KO mice than in the WT mice after correcting for multiple comparisons at the cluster-level threshold (p < 0.05). WT > *Shank3*-KO: Areas that showed significantly higher densities of neural cells in the WT mice than in the *Shank3*-KO mice after correcting for multiple comparisons at the cluster-level threshold (p < 0.05). Pixel-by-pixel t-tests were applied between two groups.

**Supplementary Table 1. Results of WT mice.**

|  | Sync | Async | p |  | Real |  | IHC |  |
| --- | --- | --- | --- | --- | --- | --- | --- | --- |
|  |  |  |  |  |  |  |  |  |
| #1 | 0.55 | 0.48 | 0.39 |  | - |  | + |  |
| #2 | 0.26 | 0.22 | 0.48 |  | - |  | + |  |
| #3 | 0.67 | 0.52 | 0.019 |  | 0.63 |  | + |  |
| #4 | 0.79 | 0.75 | 0.56 |  | 0.68 |  | + |  |
| #5 | 0.49 | 0.62 | 0.07 |  | 0.58 |  | + |  |
| #6 | 0.61 | 0.38 | 0.0020 |  | 0.44 |  | + |  |
| #7 | 0.75 | 0.62 | 0.07 |  | 0.53 |  | + |  |
| #8 | 0.68 | 0.67 | 0.45 |  | 0.85 |  | - |  |
| #9 | 0.51 | 0.44 | 0.28 |  | 0.80 |  | + |  |
| #10 | 0.72 | 0.70 | 0.75 |  | 0.53 |  | + |  |
| #11 | 0.37 | 0.32 | 0.53 |  | 0.45 |  | + |  |
| #12 | 0.52 | 0.49 | 0.61 |  | 0.69 |  | + |  |
| #13 | 0.58 | 0.42 | 0.033 |  | 0.75 |  | + |  |
|  |  |  |  |  |  |  |  |  |
|  |  |  |  |  |  |  |  |  |

Each value indicates the response rate in each subject. Sync: Response rate in the Synchronous condition. Async: Response rate in the Asynchronous condition. *p*: p-values for differences between response rates in the Synchronous and Asynchronous condition in each subject (paired t-test, Experiment 1). Real: Response rate in the real tail grasping task (Experiment 2). IHC: Immunohistochemistry.

**Supplementary Table 2. Results of Shank3-KO mice**

|  | Sync | Async | p |  | Real |  | IHC |  |
| --- | --- | --- | --- | --- | --- | --- | --- | --- |
|  |  |  |  |  |  |  |  |  |
| #1 | 0.39 | 0.50 | 0.12 |  | 0.62 |  | + |  |
| #2 | 0.25 | 0.26 | 0.84 |  | 0.58 |  | + |  |
| #3 | 0.43 | 0.36 | 0.32 |  | 0.55 |  | + |  |
| #4 | 0.18 | 0.20 | 0.72 |  | 0.38 |  | + |  |
| #5 | 0.38 | 0.29 | 0.18 |  | 0.39 |  | + |  |
| #6 | 0.42 | 0.42 | 0.97 |  | 0.55 |  | - |  |
| #7 | 0.23 | 0.18 | 0.37 |  | 0.35 |  | - |  |
| #8 | 0.17 | 0.15 | 0.77 |  | 0.41 |  | + |  |
| #9 | 0.12 | 0.11 | 0.76 |  | 0.63 |  | + |  |
| #10 | 0.11 | 0.10 | 0.71 |  | - |  | - |  |
| #11 | 0.16 | 0.13 | 0.49 |  | - |  | - |  |
| #12 | 0.30 | 0.18 | 0.014 |  | 0.45 |  | + |  |
|  |  |  |  |  |  |  |  |  |
|  |  |  |  |  |  |  |  |  |

Each value indicates the response rate in each subject.

**Supplementary Table 3. Results from dividing into first-half and second-half days (WT mice)**

|  | Sync | |  | Async | |  | Real | |  |
| --- | --- | --- | --- | --- | --- | --- | --- | --- | --- |
|  | 1st | 2nd |  | 1st | 2nd |  | 1st | 2nd |  |
|  |  |  |  |  |  |  |  |  |  |
| #1 | 0.51 | 0.58 |  | 0.38 | 0.61 |  | - | - |  |
| #2 | 0.24 | 0.29 |  | 0.20 | 0.24 |  | - | - |  |
| #3 | 0.69 | 0.66 |  | 0.55 | 0.55 |  | 0.59 | 0.67 |  |
| #4 | 0.75 | 0.84 |  | 0.67 | 0.83 |  | 0.81 | 0.54 |  |
| #5 | 0.52 | 0.47 |  | 0.62 | 0.62 |  | 0.62 | 0.55 |  |
| #6 | 0.63 | 0.60 |  | 0.42 | 0.33 |  | 0.33 | 0.57 |  |
| #7 | 0.64 | 0.85 |  | 0.64 | 0.59 |  | 0.43 | 0.63 |  |
| #8 | 0.63 | 0.72 |  | 0.69 | 0.66 |  | 0.75 | 0.97 |  |
| #9 | 0.52 | 0.49 |  | 0.48 | 0.39 |  | 0.72 | 0.88 |  |
| #10 | 0.73 | 0.72 |  | 0.70 | 0.71 |  | 0.50 | 0.56 |  |
| #11 | 0.45 | 0.26 |  | 0.32 | 0.32 |  | 0.41 | 0.50 |  |
| #12 | 0.55 | 0.48 |  | 0.42 | 0.57 |  | 0.60 | 0.78 |  |
| #13 | 0.68 | 0.45 |  | 0.50 | 0.32 |  | 0.79 | 0.72 |  |
|  |  |  |  |  |  |  |  |  |  |
|  |  |  |  |  |  |  |  |  |  |

**Supplementary Table 4. Results from dividing into first-half and second-half days (*Shank3*-KO mice)**

|  | Sync | |  | Async | |  | Real | |  |
| --- | --- | --- | --- | --- | --- | --- | --- | --- | --- |
|  | 1st | 2nd |  | 1st | 2nd |  | 1st | 2nd |  |
|  |  |  |  |  |  |  |  |  |  |
| #1 | 0.25 | 0.54 |  | 0.46 | 0.55 |  | 0.61 | 0.63 |  |
| #2 | 0.19 | 0.31 |  | 0.24 | 0.28 |  | 0.48 | 0.67 |  |
| #3 | 0.40 | 0.46 |  | 0.42 | 0.30 |  | 0.61 | 0.50 |  |
| #4 | 0.12 | 0.24 |  | 0.13 | 0.26 |  | 0.38 | 0.38 |  |
| #5 | 0.34 | 0.43 |  | 0.19 | 0.41 |  | 0.11 | 0.64 |  |
| #6 | 0.44 | 0.39 |  | 0.46 | 0.38 |  | 0.48 | 0.63 |  |
| #7 | 0.23 | 0.23 |  | 0.18 | 0.19 |  | 0.28 | 0.42 |  |
| #8 | 0.17 | 0.16 |  | 0.22 | 0.07 |  | 0.33 | 0.48 |  |
| #9 | 0.15 | 0.08 |  | 0.09 | 0.14 |  | 0.39 | 0.83 |  |
| #10 | 0.16 | 0.06 |  | 0.08 | 0.13 |  | - | - |  |
| #11 | 0.17 | 0.16 |  | 0.10 | 0.16 |  | - | - |  |
| #12 | 0.34 | 0.25 |  | 0.22 | 0.14 |  | 0.39 | 0.50 |  |
|  |  |  |  |  |  |  |  |  |  |
|  |  |  |  |  |  |  |  |  |  |
